# High-frequency imagery to capture coral tissue (*Montipora capricornis*) response to environmental stress, a pilot study

**DOI:** 10.1371/journal.pone.0283042

**Published:** 2023-03-21

**Authors:** Shuaifeng Li, Liza M. Roger, Lokender Kumar, Nastassja A. Lewinski, Judith Klein-Seetharaman, Hollie M. Putnam, Jinkyu Yang

**Affiliations:** 1 Department of Aeronautics and Astronautics, University of Washington, Seattle, WA, United States of America; 2 Department of Chemical and Life Science and Engineering, Virginia Commonwealth University, Richmond, VA, United States of America; 3 Department of Physics, Colorado School of Mines, Golden, CO, United States of America; 4 Department of Chemistry, Colorado School of Mines, Golden, CO, United States of America; 5 Department of Biological Sciences, University of Rhode Island, Kingston, RI, United States of America; 6 Department of Mechanical Engineering, Seoul National University, Seoul, Republic of Korea; University Politehnica of Bucharest, ROMANIA

## Abstract

Environment stress is a major threat to the existence of coral reefs and has generated a lot of interest in the coral research community. Under the environmental stress, corals can experience tissue loss and/or the breakdown of symbiosis between the cnidarian host and its symbiotic algae causing the coral tissue to appear white as the skeleton can be seen by transparency. Image analysis is a common method used to assess tissue response under the environmental stress. However, the traditional approach is limited by the dynamic nature of the coral-algae symbiosis. Here, we observed coral tissue response in the scleractinian coral, *Montipora capricornis*, using high frequency image analysis throughout the experiment, as opposed to the typical start/end point assessment method. Color analysis reveals that the process can be divided into five stages with two critical stages according to coral tissue morphology and color ratio. We further explore changes to the morphology of individual polyps by means of the Pearson correlation coefficient and recurrence plots, where the quasi-periodic and nonstationary dynamics can be identified. The recurrence quantification analysis also allows the comparison between the different polyps. Our research provides a detailed visual and mathematical analysis of coral tissue response to environmental stress, which potentially shows universal applicability. Moreover, our approach provides a robust quantitative advancement for improving our insight into a suite of biotic responses in the perspective of coral health evaluation and fate prediction.

## Introduction

Reef-building corals, as keystone species, support marine tropical-subtropical ecosystems and their associated biodiversity. Corals also provide a host of ecosystem services to many marine organisms and humans [1]. However, coral cover is continuously declining due to anthropogenic activities, disease outbreaks, and mass coral bleaching events associated with rising sea surface temperatures [2–9]. For example, as ocean temperatures increase, the symbiotic relationship between corals and dinoflagellate algae breaks down. The symbiotic algae are expelled from the coral tissue, which leads to the loss of pigmentation and causes the calcium carbonate exoskeleton to be visible. If thermal stress continues, corals are unable to sustain themselves and die (tissue loss, tissue sloughing). This is a global challenge which threatens the survival of hundreds of species, not only corals, but all the associated species [5,6].

To date, qualitative and quantitative methods have been developed to monitor coral tissue response, where color is an important information. Therein, qualitative methods mostly rely on visual assessment of coral color change and visual comparison between coral color and color cards, which are convenient and easy to operate [10–12]. They can provide general information about coral color and health. However, these qualitative approaches are not appropriate to a more detailed assessment and comparative study due to the subjectivity [10,13] and the dynamic nature of biological systems such as corals [14]. With the rapid development of digital photography, quantifying color change using imagery is becoming more popular. More objective and quantitative methods have also been introduced and applicable in the laboratory and field based on RGB components and brightness proportion, which overcome the drawbacks of qualitative methods [15–20]. They can provide more detailed and accurate information about coral color change over time, which makes global study possible. Digital photography also provides the possibility to study variation in algal density continuous observation, in stark contrast with the methods of color cards which uses fewer images (before/after or single photos to assess severity). However, they rely on the relatively stable lighting and manual color correction to extract exact color.

These existing approaches possess advantages and disadvantages, but there is a lack of information of dynamics in the coral tissue loss process, which hinders the future modeling. Besides, a systematic quantification with high temporal resolution at the level of the individual colony is rare, due to challenges of high-frequency continuous observation and computational demands of image processing. To fully capture the dynamics of a system, the sampling frequency needs to be properly selected. Since the frequency of coral tissue motions of *Montipora capricornis* was identified to range from 0 min^-1^ to 0.25 min^-1^, we chose to capture images of the coral every two minutes in our experiment [21]. For experimental studies, inconsistencies in lighting conditions in the underwater environment and depth can cause heavy color distortions of the captured images, which poses challenges related to color analysis and identification. The analysis of coral heath using tissue response would benefit from advanced computational approaches with universal applicability provided through color correction algorithms and the application of dynamic approaches for assessing high frequency imagery.

In biology, many organisms show signs of stochastic behavior (i.e. with a certain level of randomness or uncertainty) at different levels, from organs function (brain signals) to individual organism itself [22,23]. Corals also display stochastic behaviors in relation to tissue motion and polyp motion [21,23]. The corresponding temporal dynamics can be characterized by several measures, such as spatial distance and correlation. Therein, recurrence plots and recurrence quantification analysis have proven useful in characterizing the behavior of dynamical systems, which can be homogeneous, periodic and disrupted. Recurrence analysis can be used as a visual aid in various fields, from cognitive science [24–26], astrophysics and geophysics [27–29], damage quantification and detection [30–32], to biomedical fields [33–35]. In a similar vein, the application of recurrence plots to the analysis of coral tissue response can further our understanding of the biophysical mechanisms involved in this complex process.

Here, we observed a small colony of *Montipora capricornis* continuously over a period of one hundred days to record and investigate its tissue loss process using time-lapse imaging. We first analyzed and quantified the change of coral tissue color. As a result, we identified five stages related to changes in tissue color and morphology. Next, we tracked three polyps through time using the recurrence plot method and recurrence quantification analysis to demonstrate the dynamics of the morphology change (Pearson correlation coefficient). Notably, the recurrence analysis showed quasi-periodic and nonstationary morphology variation of the polyps. The two different approaches, one based on color analysis and the other using recurrence plots, successfully detected the critical transitions and drastic morphology change during our experimental period in a consistent and efficient manner. Here, we define “critical transition” as the stages 2 and 3. The reason will be explained later. Analyzing coral behavior qualitatively and quantitatively will open a new avenue to describe the response of coral tissue to environmental stress and its severity levels.

## Materials and methods

### Aquarium maintenance

The basic aquarium setup was similar to the setup in this reference [21]. A colony of *Montipora capricornis* (around 15 mm × 20 mm) bought from a local store was raised in a 11.36 L aquarium (37.1 cm × 22.4 cm × 28.4 cm) with specific gravity of artificial seawater = 1.024 and pH = 8.4 [21]. Artificial seawater was made from Instant Ocean Reef Crystals Reef Salt. The coral was not fed during the entire experiment. A one third water change was carried out every 3 days to maintain steady aquarium conditions. Regular tests on pH, ${NH}_{4}^{+}$, ${NO}_{2}^{-}$ and ${NO}_{3}^{-}$ were conducted using the API^©^ Saltwater Master Test kit whose resolutions are 0.25 ppm, 0.25 ppm and 5.0 ppm, respectively. The concentrations of ${NH}_{4}^{+}$, ${NO}_{2}^{-}$ and ${NO}_{3}^{-}$ were nearly zero during the whole process. The continuous water flow within the tank was provided by the Hydor Koralia Nano Aquarium Circulation Pump with 908.5 L per hour flow rate. The temperature of the water was controlled by a 50-Watt FREESEA submersible heater with the internal thermostat and ranged between 26°C- 32°C (S1 Fig). The rate of temperature increase was around 1°C every 16 days. Light was provided using a 6-Watt NICREW ClassicLED aquarium light and the ceiling light in the laboratory. Both lights were turned on (24h light cycle) during the entire experiment with a photosynthetic photon flux density (PPFD) of around 9.94 μmol**⋅**m^-2^**⋅**s^-1^ which is similar to that measured in the local coral store (~12 μmol**⋅**m^-2^**⋅**s^-1^). This measurement was done using the Photone application on iOS 15 at the surface of the water.

### Picture acquisition

We used a Canon EOS 5D Mark IV camera equipped with an EF 100mm f/2.8L Macro IS USM lens and the timer option to automatically take pictures of the *Montipora capricornis* every 2 minutes over a period of 105 days. The frequency of taking images is determined by the frequency of tissues motions of *Montipora capricornis* [21]. The parameters for the camera were: F11, ISO4000, 1/10s. The camera was mounted to a tripod for stability over the long data collection period. The macro lens was perpendicular to the wall of the aquarium to avoid the blurred effect caused by refraction. Besides, the aquarium wall was cleaned regularly to ensure the quality of images. The distance between the macro lens and the wall of the aquarium was 12.5 cm.

### Color correction and extraction of dominant colors

**Fig 1:**
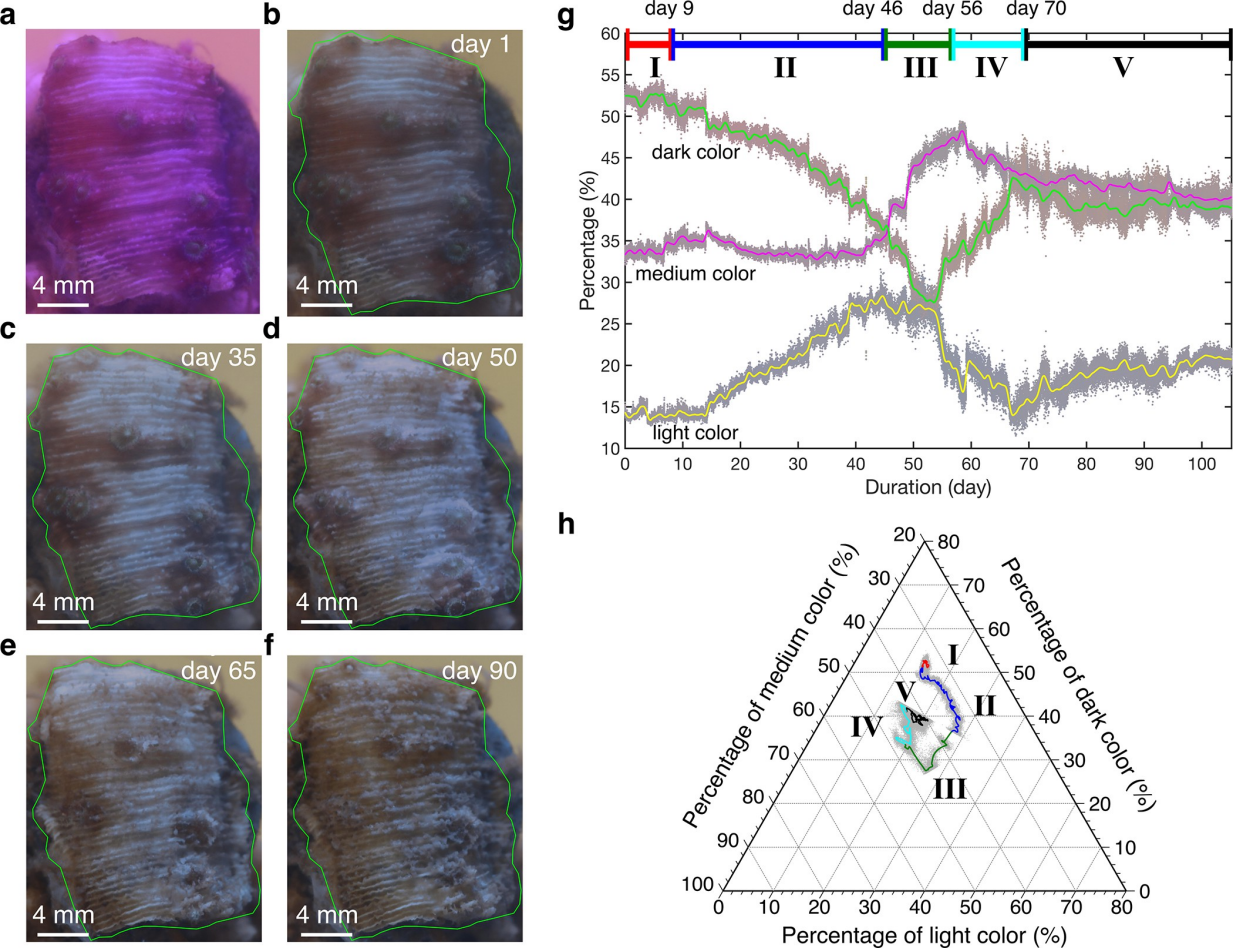
Color variation over time. **(**a**)** The image of *Montipora capricornis* on day 1 without color correction. (b)-(f) Corrected pictures of *Montipora capricornis* on days 1, 35, 50, 65 and 90, respectively. Green lines enclose the coral surface for analysis. (g**)** Variations in dominant color percentage of the coral surface over time. It includes the percentages of dark color, medium color and light color, which are plotted by dots using their colors. The smoothed variations of dark, medium and light color are represented by green, magenta and yellow lines. Different stages of coral color are shown on the top of (g**)**. (h**)** Trajectory of the color percentage change in the ternary plot. Different stages are shown by lines with corresponding colors in (g**)**.

We used the image processing algorithm to adjust the white balance of pictures imposed by the auto white balance of the camera to recover the original color without the effect of blue light. Without the color correction, the dominant colors result in the blue tint corresponding to the aquarium lights. The method was put forward in the reference [36]. It was enabled by a dataset composed of over 65000 pairs of incorrectly white balanced images and their corresponding correctly white-balanced image. Using this dataset, the k-nearest neighbor strategy was capable of computing a nonlinear color mapping function to correct the images’ colors. This method was also verified for pictures not in the training set. This color correction method is preferred when color reference cards are not available or difficult to be captured in the frame. By applying this algorithm to the original picture (Fig 1(A)), it became the corrected picture (Fig 1(B)). The picture was similar to what the coral looked like under natural light. This method is applied to all images to ensure the same baseline (images taken under the natural light) to perform the image analysis. After correcting the color, three dominant colors were extracted from the coral surface (region of interest enclosed by the green line, Fig 1). Three channels (Red channel, Green channel and Blue channel) were vectorized and clustered into three groups in RGB space using the k-means algorithm based on the minimal Euclidean distance, resulting in three dominant colors sorted by the percentage. Note that these three clusters are chosen for physical interpretation of light, medium and dark colors instead of minimizing the cost function of k-means clustering. The color with the highest proportion represented the first dominant color, and so on. Since these three clusters were distributed in RGB color space with certain distance, visually the shades of color were distinct and were composed of distinct RGB values, which would be used as light, medium and dark colors in our analysis.

### Calculation of pearson correlation coefficient

The coral tissue response under environmental stress includes not only the evolution of the dominant colors, but also the morphology change over time. Here we chose three polyps on the coral surface to study the dynamics of morphology. Fig 1(B) shows a picture of the coral colony and three polyps on its surface, named polyp A, polyp B and polyp C. To depict the evolution of the polyp, we introduced the intensity plot that indicated the image similarity between the reference and the current images based on image correlation [37,38]. Intensity plots were constructed by plotting the pixel values of the green channel in the reference image along the *x*-axis, and the intensity value of the same pixel location in the current image on the *y*-axis, thereby forming a bivariate histogram that describes the relationship between the corresponding intensity values. The green channel was chosen for the intensity plots due to the stability in three dominant colors compared with other channels (see the S2 and S3 Figs). This allowed us to obtain the morphology change instead of the light variation. Note that we analyzed images with color correction.

To study the dynamics of the polyps’ similarity, we used the intensity plots and adopt the Pearson correlation coefficient to calculate the variation of these intensity plots over the whole period. This can be calculated as follow:

$$r_{t}=\frac{\sum_{m}\sum_{n}\left(I_{mn}^{r}-\bar{I^{r}})(I_{t,mn}-\bar{I_{t}}\right)}{\sqrt{\sum_{m}\sum_{n}{\left(I_{mn}^{r}-\bar{I^{r}}\right)}^{2}\sum_{m}\sum_{n}{\left(I_{t,mn}-\bar{I_{t}}\right)}^{2}}}$$

where *I*^*r*^ represents the intensity value of the reference picture (i.e., picture at day 1) and *I*_*t*_ represents the intensity value of the picture at day *t*, and *m*, *n* represent the pixel index. $\bar{I^{r}}$ and $\bar{I_{t}}$ are the mean intensity values of the reference picture and the picture at day *t*, respectively. This method combined with normalization of pixel intensity can minimize the effect of brightness increase due to water level decrease.

### Calculation of recurrence plot and recurrence quantities

Dynamical systems have the essential characteristic of recurrence, which may be used to describe the system’s behavior in phase space [39,40]. We applied this recurrence concept to our coral system based on the correlation coefficients calculated above. A powerful tool for the visualization and analysis of recurrence is called a recurrence plot, which is mathematically expressed as follows:

$$R_{ij}=\Theta (\varepsilon -\|\vec{r_{i}}-\vec{r_{j}}\|),\vec{r_{i}}\in R^{m},i,j=1,2,\ldots ,N$$

where *N* is the number of considered states *r*_*i*_ (i.e., *N* is the number of pictures, and *r*_*i*_ is the Pearson correlation coefficient for the day *i*), *ε* is a threshold distance, ‖∙‖ is the norm (the Euclidean norm) and *Θ*(∙) is the Heaviside function. Here the threshold distance *ε* was chosen to be 0.02. When *r*_*i*_ is in proximity to *r*_*j*_ within the threshold distance *ε*, the Heaviside function is one and shown in red on the recurrence plot. Otherwise, it is zero and shown in white on the recurrence plot. The recurrence plot obtained from the correlation coefficient allows a visual interpretation of the coral’s dynamic morphology change from the perspective of similarity compared with the initial stage, thus characterizing coral tissue response, polyps death and macroalgae overgrowth graphically, which is a useful addition to the color analysis.

Recurrence quantification analysis was used to characterize the dynamics of morphology change, where we calculate the recurrence rate (*RR*), determinism (*DET*), longest diagonal line (*LMAX*), entropy (*ENT*), laminarity (*LAM*) and trapping time (*TT*). In addition, we compared these quantities within different polyps and different stages. Recurrence rate (RR) measures the probability that a concrete state will recur and is mathematically expressed as follows:

$$RR=\frac{1}{N^{2}}\sum_{i,j=1}^{N}R_{ij}$$

Apart from the measures based on recurrence density, measures based on diagonal lines including the main diagonal line and parallel diagonal line can also characterize the complexity of the system. Determinism (DET) is the percentage of recurrence points that form diagonal lines expressed as:

$$DET=\frac{\sum_{l=l_{min}}^{N}lP(l)}{\sum_{l=1}^{N}lP(l)}$$

where *P*(*l*) is the histogram of the lengths of the diagonal lines *l* (see S4 Fig), measuring the predictability of the system. Deterministic processes produce longer diagonal and fewer single, isolated recurrence points than processes with uncorrelated or weakly correlated, stochastic behaviors.

Another important quantity is *LMAX* which measures the length of the longest diagonal line segment in the recurrence plot, excluding the main diagonal line expressed as:

$$LMAX=\max\left(\left\{l_{i};i=1,\ldots ,N\right\}\right)$$

It is inversely related to the largest positive Lyapunov exponent with complex relation [41,42]. The measure of the complexity of the recurrence plot with respect to the diagonal lines by Shannon entropy (*ENT*) can be mathematically expressed as:

$$ENT=-\sum_{l=l_{min}}^{N}p(l)\ln p(l)$$

where $p(l)=\frac{P(l)}{\sum_{l=l_{min}}^{N}P(l)}$ can estimate the probability that a diagonal line has exactly length *l*.

The recurrence plot can also be measured by the vertical lines. Analogous to determinism, laminarity (LAM) describes the percentage of recurrence points that form vertical lines expressed as:

$$LAM=\frac{\sum_{v=v_{min}}^{N}vP(v)}{\sum_{v=1}^{N}vP(v)}$$

where *P*(*v*) is the histogram of the lengths of the vertical lines *v* (S5 Fig), measuring the occurrence of laminar states in the system. Another important measure regarding the vertical lines is trapping time (TT), which reflects the average length of the vertical lines:

$$TT=\frac{\sum_{v=v_{min}}^{N}vP(v)}{\sum_{v=v_{min}}^{N}P(v)}$$

This parameter informs us for how long the system is trapped in a specific state. It represents frequency and length of laminar states.

## Results

### Color variation during observation period

We recorded the dynamic process of tissue response and macroalgae overgrowth of *Montipora capricornis* under thermal stress (26°C to 32°C) and irradiation stress over a period of 105 days (see the time lapse video in the S1 Movie and temperature in the S1 Fig). Fig 1(A) exhibits the captured picture of *Montipora capricornis* on day 1. As an example, the corresponding corrected picture on day 1 is shown in Fig 1(B). The RGB histograms of the original picture and the corrected picture on day 1 are exhibited in S2 Fig. After correction, the green components did not change much but the red and blue components changed from large to small values. Changes in both color and morphology were observed through time (Fig 1(B)–1(F)). On day 1, the coral tissue appeared smooth and tight over the surface, while at day 90 the tissue appeared to detach from the coral skeleton and the macroalgae overgrew the skeleton surface. Moreover, the color variation was quite complicated with shifts from pale to dark at different moments in time. Therefore, we need to trace and analyze the color variation of these corrected images throughout the entire experimental period.

We focused on the coral tissue area outlined in green (Fig 1(B)), and the three dominant colors of that area were extracted using the K-means algorithm in the RGB color space [43] as described in the Materials and methods. From bottom to top, there were three extracted colors–light, medium and dark–plotted by using their own colors but represented by yellow, magenta and green lines after smoothing for visualization purposes (Fig 1(G)). The raw RGB components of three dominant colors also show the evolution (S3 Fig). It should be noted that the RGB components do not change significantly so that the centers of three clusters are located in a narrow region in the RGB color space.

By comparing the variations of the relative percentages of the three dominant colors during the time-lapse video (S1 Movie) we detected four inflection points which revealed a 5-stage process (see bars in Fig 1(G)). In the first stage (days 1–9), the dark color was dominant (>50%) while the medium color and light color were the second and third dominant colors, respectively (~34% and ~14%, respectively). The coral was in a healthy state and the percentages of the three dominant colors barely changed. In the second stage (days 9–46), the coral surface suffers a visible change. The area at the top center of the coral becomes paler, with this phenomenon (tissue loss) spreading laterally. This lateral spread is characteristic of tissue loss as opposed to bleaching. The proportion of dark color started to decrease (-15% compared to day 1), and of the proportion of medium color had a slight increase. At day 15, the proportion of light color started to increase (+13% compared to day 1). The coral on day 1 and the coral on day 35, however, did not exhibit the significant change on polyps integrity (Fig 1(B) and 1(C)). At around day 40, the tissues on the coral surface started to experience tissue loss in a large area, suggesting that the tissue integrity is extremely compromised (S1 Movie) [44]. At day 46, the proportion of light color reached the peak (~30%). This is due to the major tissue loss making the coral more vulnerable to death. This is a critical stage because only the polyps remain. If environmental conditions return to normal, one can assume the polyps will extend their connective tissue allowing the coral fragment to recover with time (also assuming low algal competition). In the third stage (days 46–56), the proportion of dark color continued to decline (-4% compared to day 46) and that of medium color began to increase (+10% compared to day 46). The medium color became the first dominant color in this stage. Meanwhile, the proportion of light color almost remained constant and decreased in the end. The pale coral became paler, resulting in the exposure of the coral skeleton (Fig 1(D)). Moreover, according to the time-lapse video, the polyps did not show movement anymore, indicating the main polyps have receded into the skeleton. In the fourth stage (days 56–70), the proportion of light color declined (-4% compared to day 56) while that of dark color increased gradually (+8% compared to day 56). The macroalgae gradually grew on the coral surface as seen in Fig 1(E). The fifth stage (days 70–115) marked a stabilization of the relative percentages of dominant colors due to the macroalgae overgrowth covering the fully exposed coral skeleton. Although the last two stages are observed during our experiment, they are not directly related to the process of the coral tissue response. The trend of the quantitative color variation (increasing percentage of light color and decreasing percentage of dark color) agrees well with the qualitative results in the previous study [10,19,20].

To trace the trajectory of the three dominant colors, the variation of their percentage was shown by gray dots in the ternary plot (Fig 1(H)). After smoothing, the trajectory of color variation was exhibited in different colors corresponding to different stages. The variation of color moved clockwise to form a trajectory that is similar to an open ring. However, the data points eventually converged to the approximate center of the ring, which has around 38.93%, 40.30% and 20.77% for the dark, medium, and light colors. It will be of great interest to explore whether this asymptotic destination is unique or variant depending on the coral species and/or their environmental factors.

### Pearson correlation coefficient analysis

**Fig 2:**
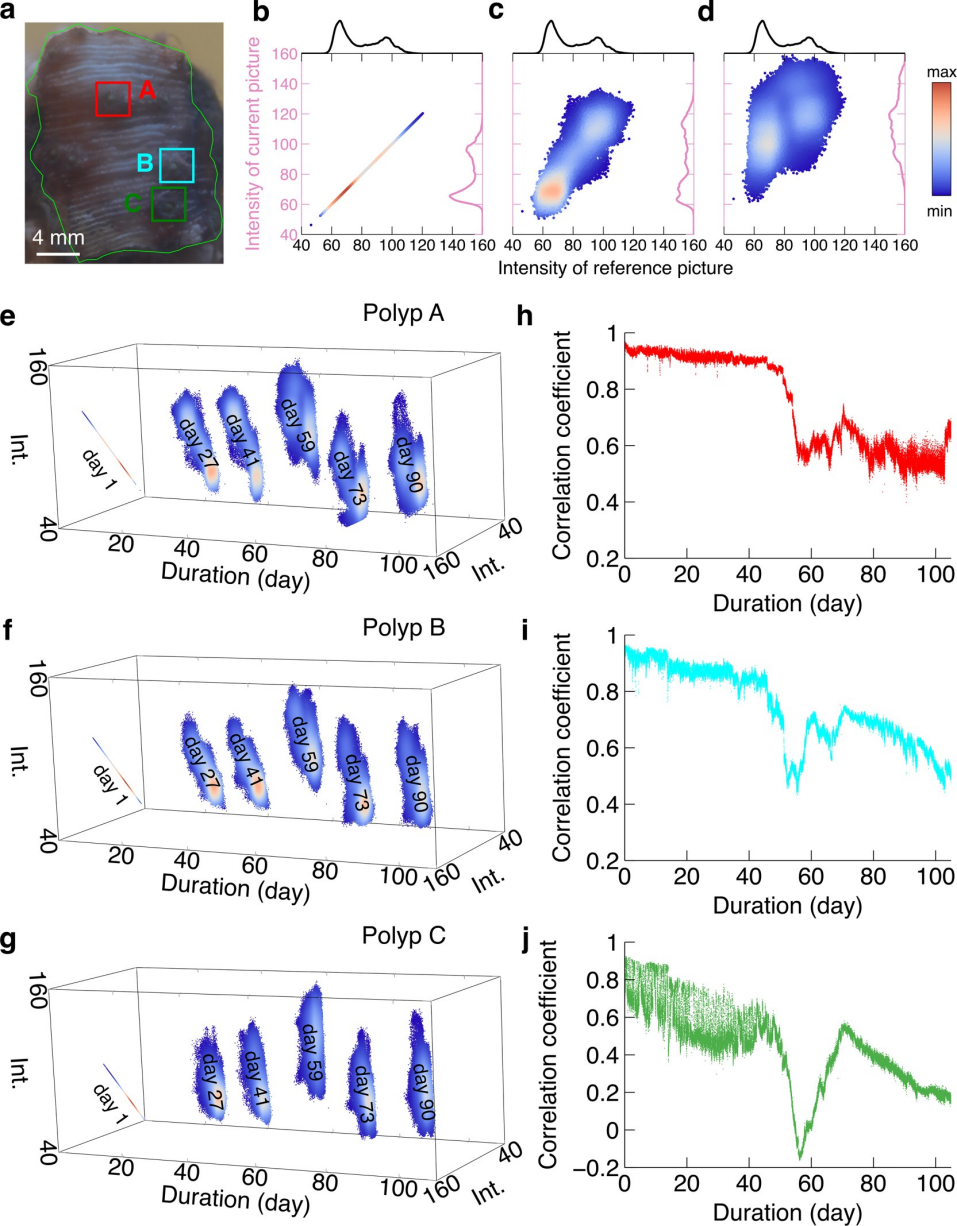
Intensity plot and Pearson correlation coefficient. (a). The image of *Montipora capricornis* on day 1 with color correction. Polyps A, B and C are shown in red, cyan and green. The intensity plot of the green channel of polyp A on (b) day 1. (c) day 27, and (d) day 59. The histograms of the reference picture and current picture are shown on the top in black and on the right in magenta. Intensity plots of green channels of polyp A sliced on days 1, 27, 41, 59, 73 and 90 are shown for (e) polyp A, (f) polyp B, and (g) polyp C. The density is encoded with color. Pearson correlation coefficients as a function of time when taking the first picture as the reference for (h) polyp A, (i) polyp B, and (j) polyp C.

Fig 2(A)–2(C) showed the intensity plots between the reference picture (day 1, represented by the *x*-axis) and the pictures at days 1, 27 and 59 (*y*-axis) of polyp A. Note that the pixel values of the green channel of the corrected images are used as intensity. The histograms of the reference picture and the current picture are shown on the top and on the right of the graph. The density of the scatters was encoded by the color to represent the 2D histogram. A narrow diagonal distribution implies a stronger correlation (i.e., less morphology change), while a wide scattered distribution means a weaker correlation (i.e., more drastic morphology change). Obviously, Fig 2(A) exhibits a straight line because the reference picture and the first picture are identical. However, Fig 2(B) and 2(C) show gradually wider distributions in the intensity plot, suggesting the evolution of the morphology compared with the reference picture.

We stacked several intensity plots of polyp A at days 1, 27, 41, 59, 73 and 90 (Fig 2(D)). The scatter dots became dispersed over time, suggesting that images were becoming less and less correlated, meaning that similarity between the current picture and the reference picture is decreasing. Besides, the orientations of scatter dots showed the positive correlation of the reference picture and the other pictures. The density of scatter dots also varied from high density of small values to relatively even density distribution. Similarly, the intensity plots at the same period for polyp B and polyp C had the similar trend as polyp A (Fig 2(E) and 2(F)).

The Pearson correlation coefficient reflected the similarity, revealing the dynamics of morphology. At around day 50, there were big declines in all three figures, suggesting drastic changes in polyp morphology (Fig 2(G)–2(I)). Compared with polyp A and polyp B, polyp C had the largest morphology change at around day 50 as indicated by the largest variation of correlation coefficients. We observed that most parts of the polyps became fluffy and hollow around day 50 from the raw images (see S1 Movie). Finally, because the macroalgae grow on the surface of corals, their morphology will be covered by the macroalgae. However, the similarity will not change significantly so that the Pearson correlation coefficient will converge. The finding from this Pearson correlation coefficient is consistent with the result based on the extracted dominant colors (Fig 1(G)), where we hypothesized the drastic morphology change to be death of corresponding polyps.

### Characterization of recurrence plot

**Fig 3:**
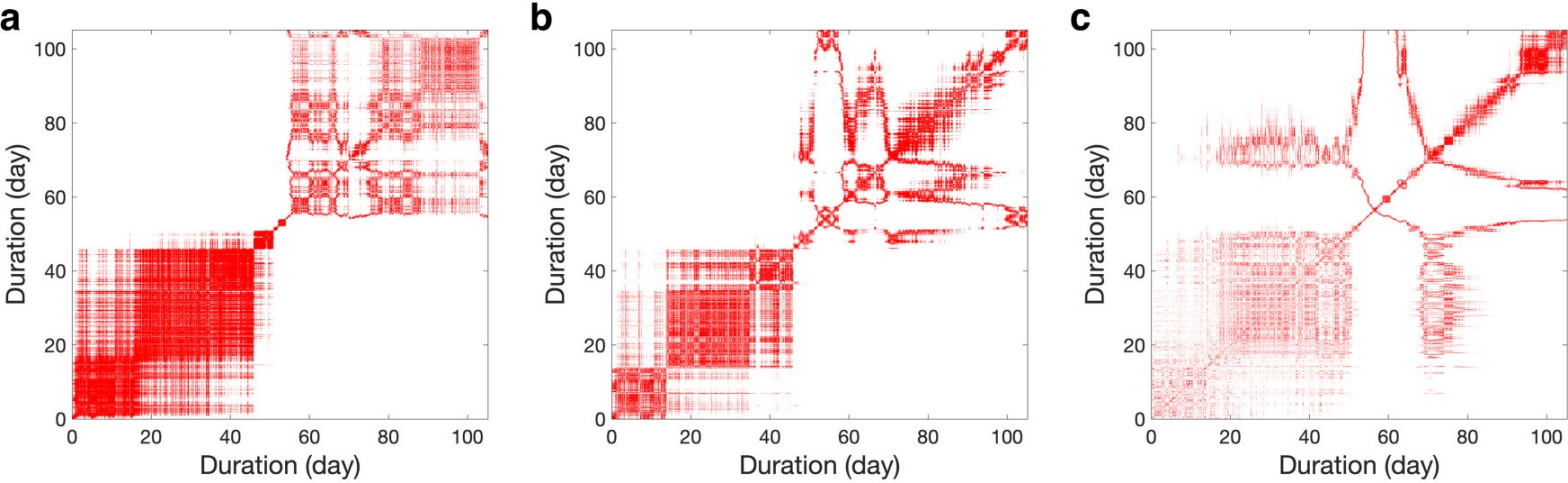
Recurrence plot. The recurrence plot of the Pearson correlation coefficients of (a) polyp A, (b) polyp B, and (c) polyp C.

We calculated the recurrence plots to compare three polyps and macroalgae overgrowth during the experiment (Fig 3(A)–3(C)). The characteristic patterns on the recurrence plot, which was denoted as typology, are graphic features to describe dynamical systems [45]. According to these three recurrence plots, characteristic typology had some similarities and differences. Upper left and lower right corners had large white areas caused by certain drift of correlation coefficients, which is the sudden decline of the correlation coefficients. The recurrence plots were composed mainly of two parts separated by an inflection point on the curves representing correlation coefficients. The inflection points on the correlation coefficient and recurrence plot correspond to the drastic morphology change in the tissue, which also corresponds to the end of the third stage of the color variation. The lower left corner represented the polyp’s behavior before possible death, where the typology was quasi-periodic with certain drift (black lines unevenly aligned in a certain pattern), indicating the dynamics of variation of morphology similarity. It was obvious that the density of lower-left pattern was high for polyp A and low for polyp C, suggesting the higher possibility for polyp A to return to the original position after a certain period. The upper right corner represented the period of macroalgae overgrowth, where disruptions occurred, suggesting the nonstationary dynamics of morphology. The swinging of detached tissue induced by water flow also contributed to the nonstationary dynamics. Besides, three polyps had distinct patterns of disruptions, which implied that macroalgae overgrowth was distinct at different locations. It should be noted that the quasi-periodic typology characteristic, similar to the lower left corner in Fig 3(A), is expected in the recurrence plot in the healthy coral.

**Fig 4:**
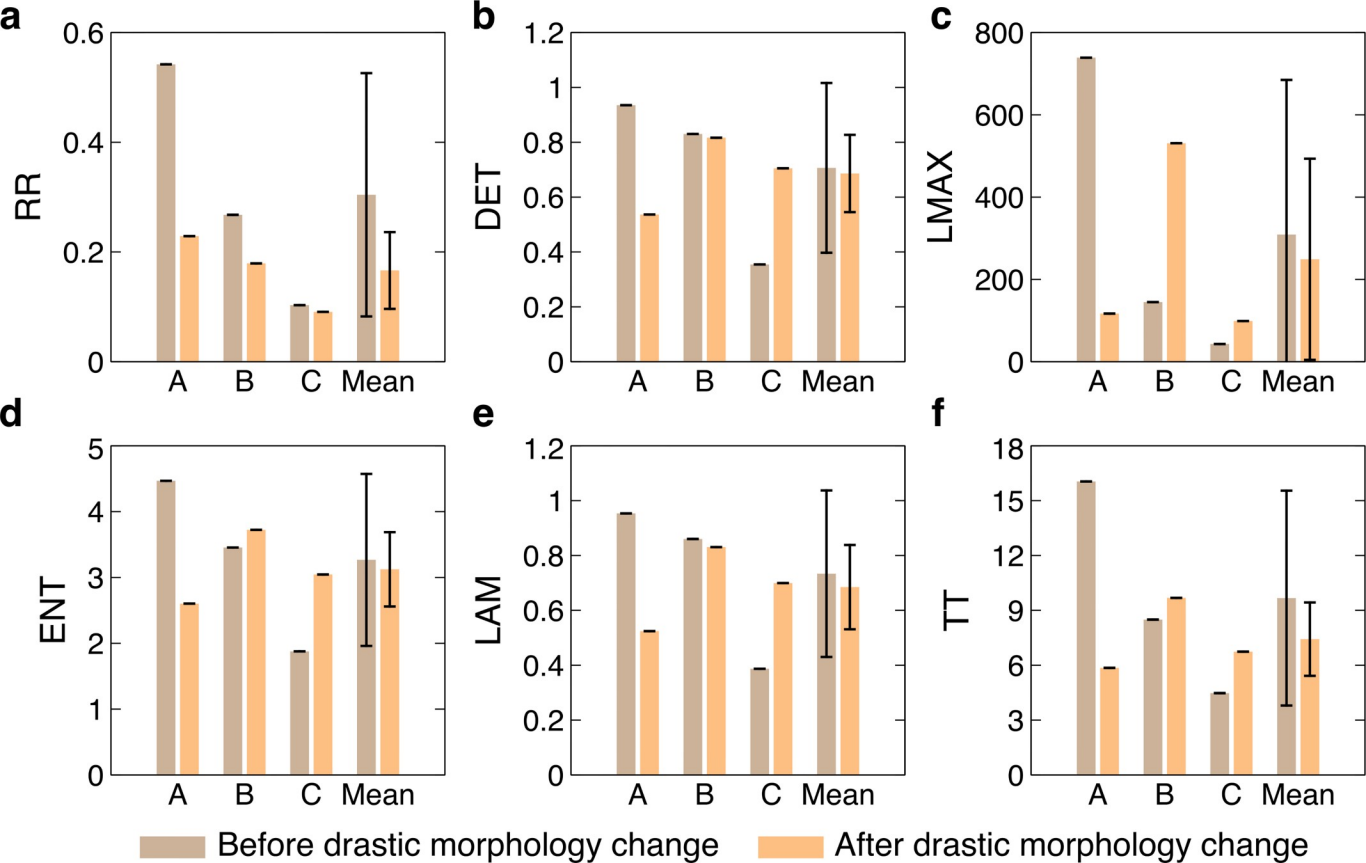
Recurrence quantification analysis. The recurrence rate (*RR*), determinism (*DET*), longest diagonal line (*LMAX*), entropy (*ENT*), laminarity (*LAM*) and trapping time (*TT*) for polyp A, polyp B and polyp C are shown in (a), (b), (c), (d), (e), (f), respectively. The means with error bars calculated by the standard deviation of corresponding quantities are also displayed. Corresponding quantities are shown in brown for the situation before the drastic morphology change and in orange for the situation after the drastic morphology change.

We conducted the recurrence quantification analysis to characterize the recurrence plot. We also compared these quantities among different polyps to discover the distinctions. The quantities before the drastic morphology change can represent the natural variance of coral polyps, while they can be unpredictable and random after the drastic morphology change. Therefore, we particularly explore the quantities before the drastic morphology change. The RRs of three polyps before and after the drastic morphology change were measured and indicate the probability that the coral morphology will return to the original state after a certain period (Fig 4(A)). Here, coral tissues motions contribute to part of the variation in morphology. Higher RR means lower system variability. Before the drastic morphology change, polyp A had the largest RR and polyp C had the smallest RR.

Larger determinism means higher deterministic system. It indicates that the behaviors of some polyps can be predictable. Polyp A had the best predictability than others before the drastic morphology change (Fig 4(B)). From the perspective of LMAX to measure the predictability, the smaller the *LMAX* is, the less stable the polyp will be. Polyp A was most stable among three polyps before the drastic morphology change (Fig 4(C)). ENT indicates the complexity of the behaviors of coral polyps. Before the drastic morphology change, polyp A had the most complex recurrence plot (Fig 4(D)). Larger LAM indicates stronger intermittency, which shared a similar trend to *DET*. Polyps A, B and C had decreasing *LAM*s before the drastic morphology change (Fig 4(E)). From the perspective of trapping time to measure intermittency, longer *TT* indicates stronger intermittency. *TT* shared a similar trend to *LAM*. Polyp A had the longest *TT* before the drastic morphology change (Fig 4(F)). The corresponding means and error bars calculated by the standard deviation of each quantity are shown in Fig 4. These quantities can potentially help with future applications on machine learning and automation to identify coral status and to model the coral tissue response.

## Discussion

Here we provide a complete time-lapse recording of the response of a small coral colony to severe environmental stress spanning 105 days. Based on this comprehensive recording, we analyzed coral tissue color variation and polyp morphology during the whole experimental period, which remained largely unexplored before. The introduction of recurrence plots and corresponding recurrence quantification analysis characterized the dynamics of polyp tissue, which revealed the quasi-periodic and nonstationary morphology change at different stages of tissue loss. Specifically, polyp morphology changes relatively regularly before the drastic morphology change, but after the drastic morphology change, it is more erratic dominated by the environment. The color- and morphology-based techniques both captured the more subtle transition to tissue decay successfully and consistently, which confirms the applicability of this approach to the study of coral tissue response to environmental change.

Compared with the previous studies [15,16], the color analysis technique presented here not only extracts the RGB components but it also identifies the dominant colors (color shade percentage), which offers a new approach to accurately quantify tissue color variation over time in response to environmental stress. It is also helpful for the evaluation of coral health and fate prediction related to the progression of tissue loss. Indeed, the second and third stages appear to be key with recovery still possible as long as polyp tissue remains.

Using the correlation analysis (Fig 2) and corresponding recurrence plot (Fig 3), we can systematically identify the drastic morphology change in the observations where the corresponding values present a large sudden decline for all three studied polyps. According to the time-lapse video and color analysis above, this drastic morphology change is likely to indicate the moment when the coral polyps die from tissue loss.

Ocean warming is amongst the most critical contemporary perturbations threatening reef-building coral survival worldwide. It is urgent to better understand the impacts of these global perturbations on coral behavior. Corals are complex organisms capable of sensing and responding to environmental conditions. Despite typically not having developed eyes, corals respond to light of different intensities and wavelengths [46]. Therefore, by leveraging our methodology, it will be inspiring to study visual responses of *Montipora capricornis* in greater detail, and to extend the work to the study of other coral species under different light intensities and wavelengths. Our approach may be extended to the more practical scenarios where multiple colonies of corals are studied in the laboratory and field for the robustness from a biology standpoint instead of the physics standpoint in our work. But several challenges need to be addressed such as the camera setting for different coral species and data processing of images taken in the dark. As for the study of multiple colonies in different tanks and experimental replicates, it is possible by multiplying the number of cameras or using one camera with a sliding rail system. Observations in the field are more challenging considering the stability requirements and the need for a clean camera lens over an extended period of time. Our methods of color correction and dominant color extraction could help with images taken under the unstable light conditions such as in the field, which would address the challenges above. The effect of a single environmental variable such as thermal stress or irradiation stress can also be explored in future studies with more laboratory experiments using our methods, since in our current experiment, there are several environmental variables including water level, turbidity, algae growth and feeding. Besides, the coral colonies in different geographical locations around the globe may have different responses to environment variables, but still more laboratory experiments are needed to understand the realistic scenes.

Compared with existing methods based on long-term recordings and color reference cards to extract color information [10,11,15–20], we focus more on the dynamics of the coral system and deliberately set the frequency of recording according to the tissue motion of *Montipora capricornis* [21]. Without the adequate frequency of recording, the trends for dominant color and correlation coefficient variations would be similar but the high-frequency information would be lost, resulting in the incorrect characterization of the dynamics of coral tissue response. However, for practical applications instead of the physical characterization, the downsampling of image series is feasible to calculate the recurrence quantities due to the scalable nature. Furthermore, this study quantitatively explores tissue morphology variations which is an innovative approach to studying the effects of environmental stress leading to coral death. Note that the dynamics of coral tissue response are complex and related to many environmental variables, as well as the relation between coral tissue response and coral physiology. This needs future detailed exploration that is enabled by the novel technology described here. The image-based approaches to analyze coral tissue respnse demonstrated in this study offer us possibilities to visualize the imperceptible dynamics of stony corals, which could boost coral biological study.

The code and corresponding data are available in the Open Science Framework DOI 10.17605/OSF.IO/V4K8G.

## Supporting information

S1 Fig The variation of temperature during the experiment. The red circles represent temperatures on certain days. The black line represents the variation of temperature with the assumption that the temperatures remain constant until the recording day. Note that the drop near day 60 was caused by technical issues with the electricity.
(TIFF)

S2 Fig Histograms of the original picture and the corrected picture. (a**)** The histogram of the first picture of *Montipora capricornis* with original color. (b) The histogram of the first picture of *Montipora capricornis* with corrected color. The histograms of red, green, blue channels are represented by red, green and blue lines, respectively.
(TIFF)

S3 Fig RGB values of three dominant colors. The RGB components of light color, medium color and dark color as a function of duration are shown from left to right for the (a). R channel, (b). G channel, and (c). B channel.
(TIFF)

S4 Fig The histograms of diagonal lines in recurrence plot. The left panel shows the histogram of diagonal lines in recurrence plot for each polyp before the drastic morphology change. The right panel shows the histogram of diagonal lines in recurrence plot for each polyp after the drastic morphology change.
(TIFF)

S5 Fig The histograms of vertical lines in recurrence plot. The left panel shows the histogram of vertical lines in recurrence plot for each polyp before the drastic morphology change. The right panel shows the histogram of vertical lines in recurrence plot for each polyp after the drastic morphology change.
(TIFF)

S1 Movie Coral tissue response of *Montipora capricornis*. This time-lapse video shows the coral tissue response of *Montipora Capricornis* in 105 days. It is played at 3000X speed playback.
(MP4)
