# Supplementary figures and images for: High-frequency imagery to capture coral tissue (Montipora capricornis) response to environmental stress, a pilot study
Source: PLoS One. 2023 Mar 21;18(3):e0283042. doi: 10.1371/journal.pone.0283042 (PMC10030036; doi:10.1371/journal.pone.0283042)

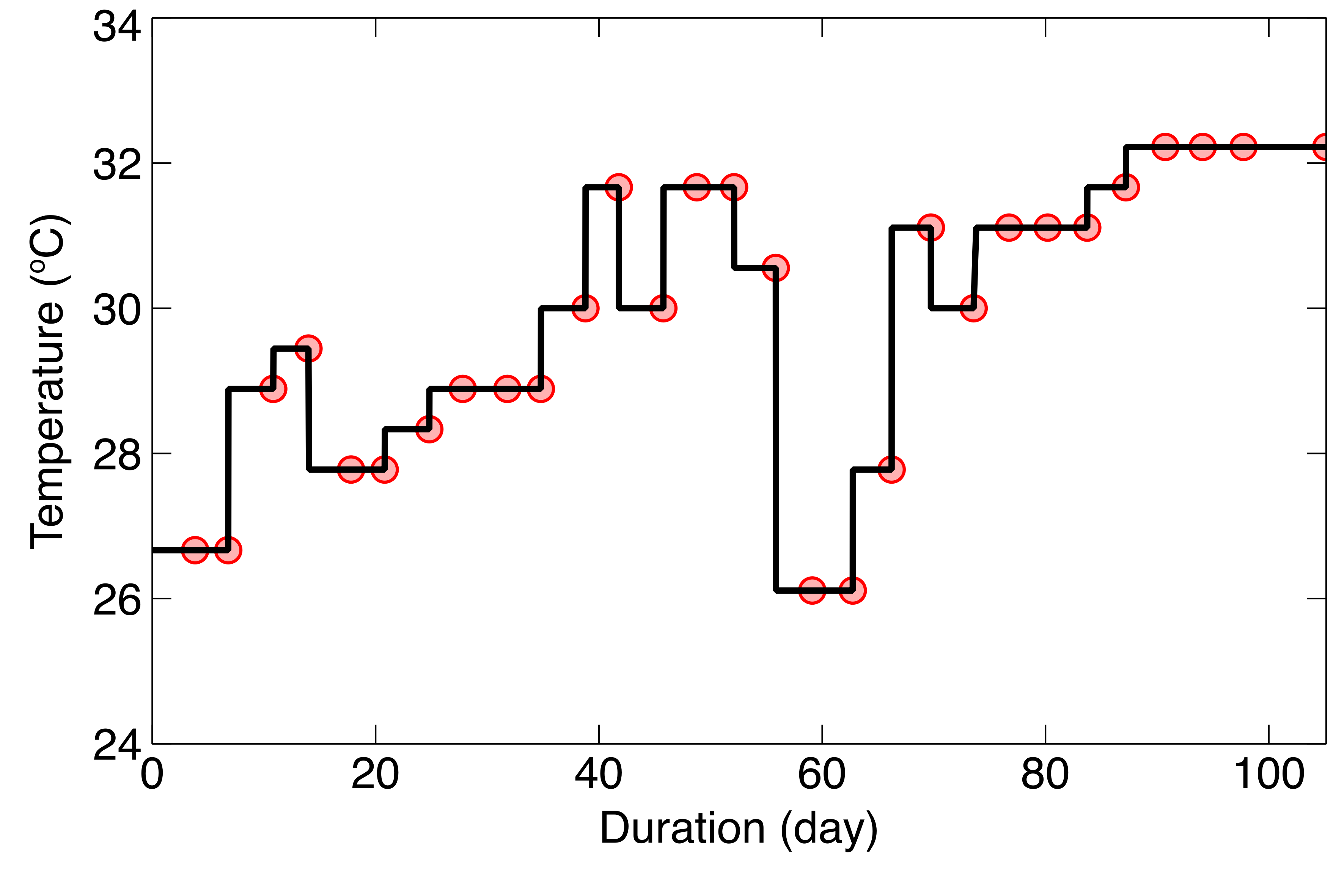

Supplement: S1 Fig — The red circles represent temperatures on certain days. The black line represents the variation of temperature with the assumption that the temperatures remain constant until the recording day. Note that the drop near day 60 was caused by technical issues with the electricity. (TIFF) [file pone.0283042.s001.tiff]

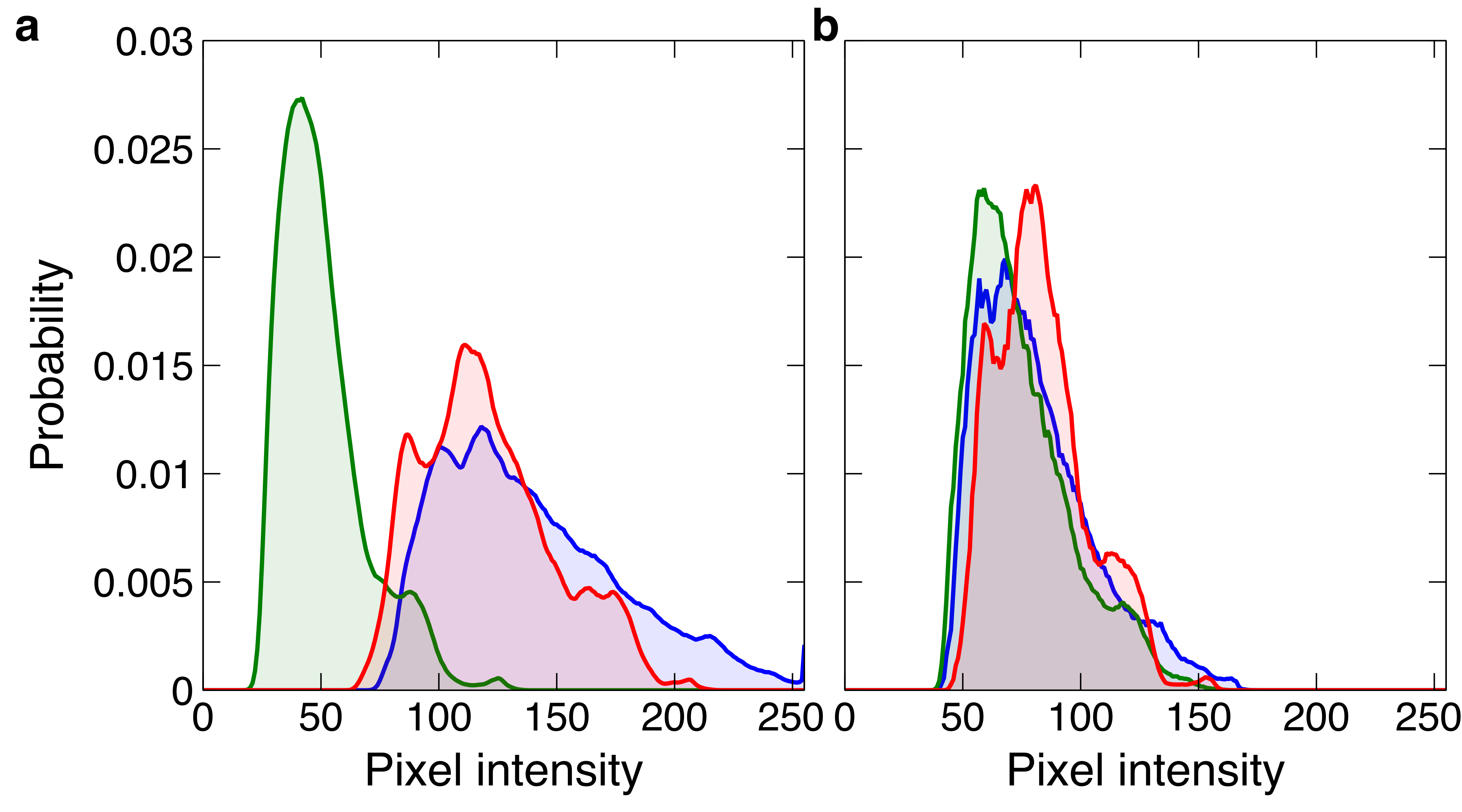

Supplement: S2 Fig — (a) The histogram of the first picture of Montipora capricornis with original color. (b) The histogram of the first picture of Montipora capricornis with corrected color. The histograms of red, green, blue channels are represented by red, green and blue lines, respectively. (TIFF) [file pone.0283042.s002.tiff]

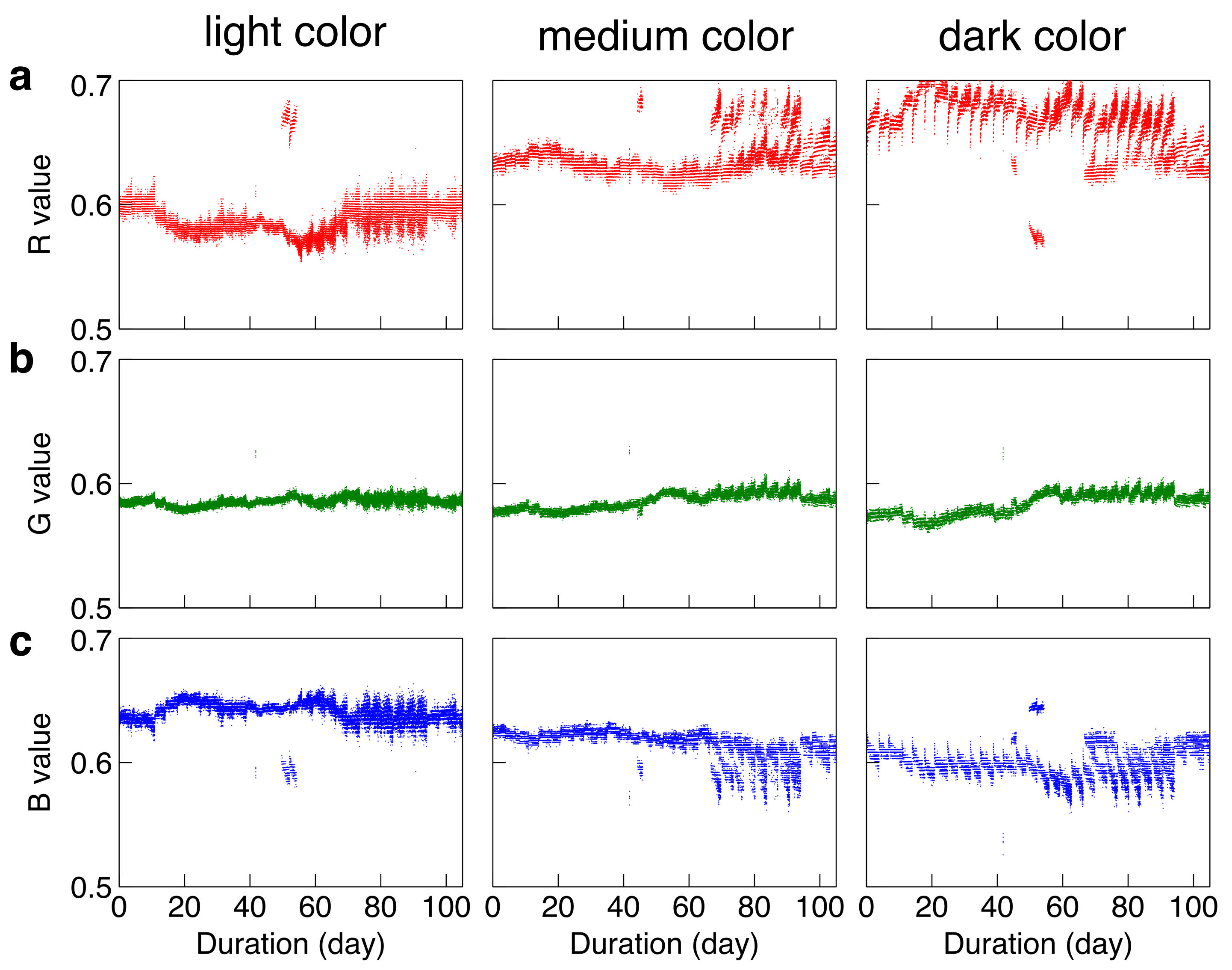

Supplement: S3 Fig — The RGB components of light color, medium color and dark color as a function of duration are shown from left to right for the (a). R channel, (b). G channel, and (c). B channel. (TIFF) [file pone.0283042.s003.tiff]

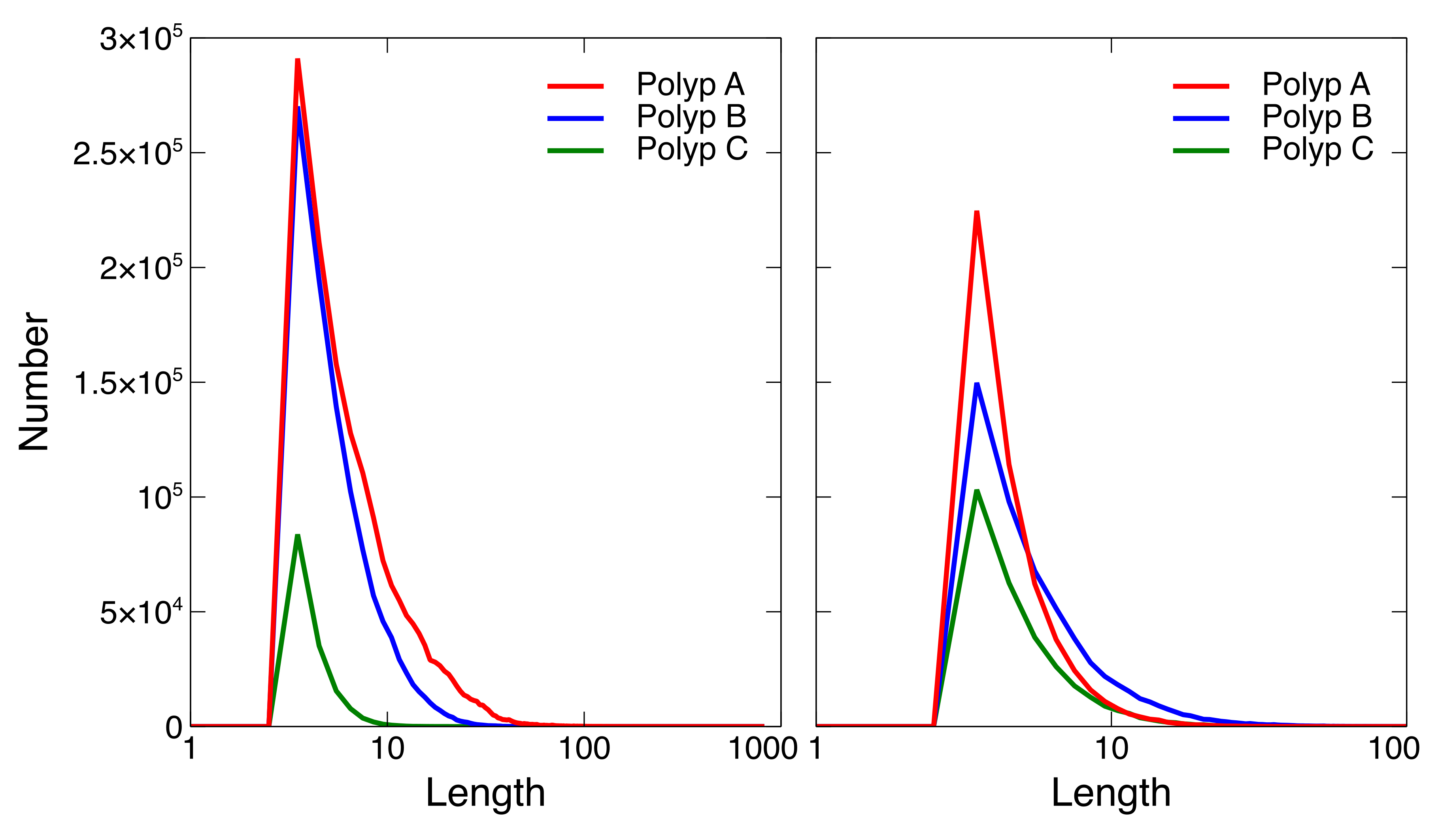

Supplement: S4 Fig — The left panel shows the histogram of diagonal lines in recurrence plot for each polyp before the drastic morphology change. The right panel shows the histogram of diagonal lines in recurrence plot for each polyp after the drastic morphology change. (TIFF) [file pone.0283042.s004.tiff]

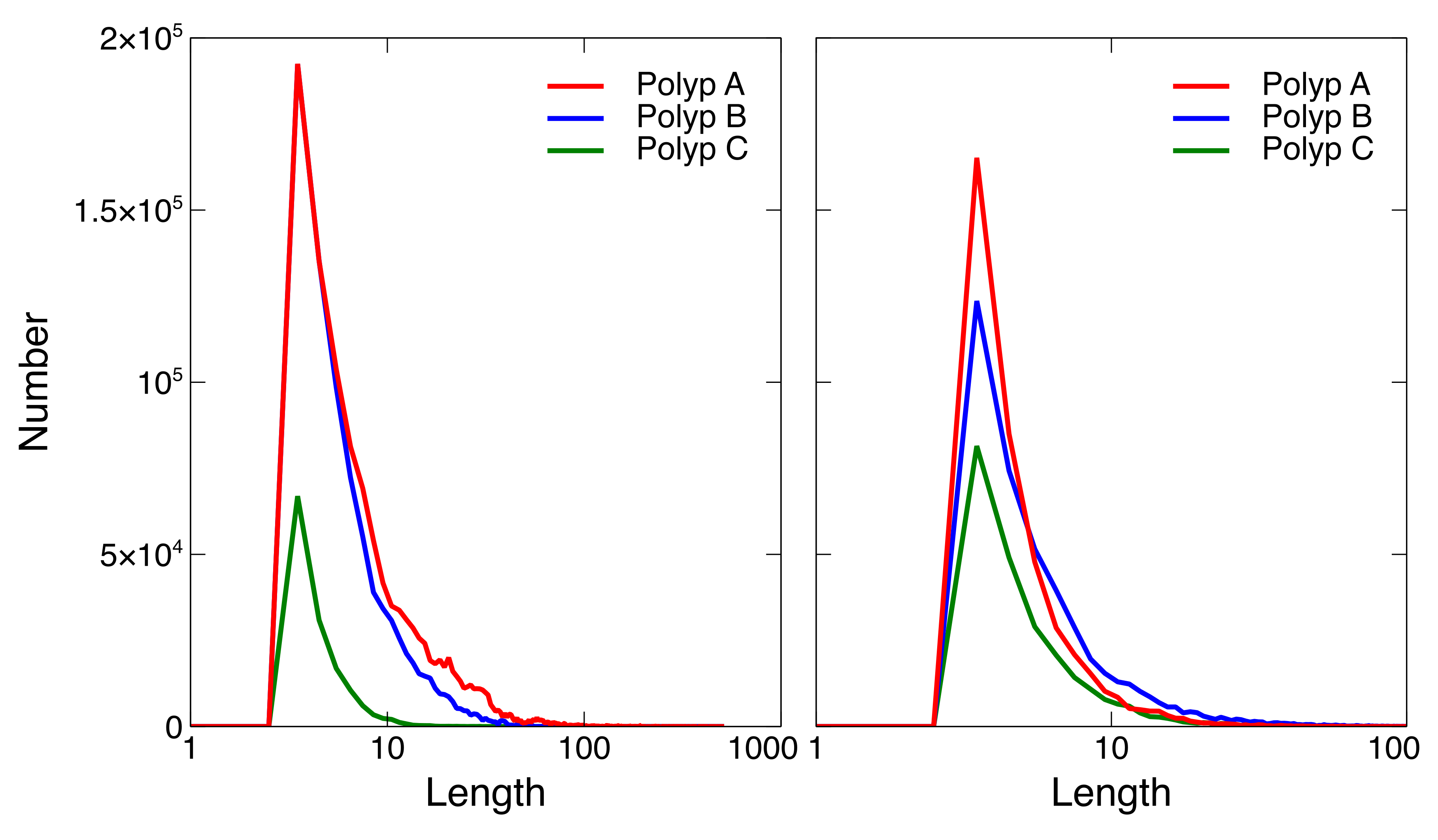

Supplement: S5 Fig — The left panel shows the histogram of vertical lines in recurrence plot for each polyp before the drastic morphology change. The right panel shows the histogram of vertical lines in recurrence plot for each polyp after the drastic morphology change. (TIFF) [file pone.0283042.s005.tiff]
